# Cancer-related fatigue in patients undergoing hematopoietic stem cell transplant: a cross-sectional study on prevalence and influencing factors

**DOI:** 10.3389/fpsyt.2025.1681573

**Published:** 2025-12-08

**Authors:** Xia Wu, Qiufang Xu

**Affiliations:** Department of Hematology, The First Affiliated Hospital of Soochow University, Suzhou, Jiangsu, China

**Keywords:** fatigue, hematopoietic stem cell transplantation, assessment, nursing, care

## Abstract

**Background:**

Fatigue constitutes a highly prevalent symptom within the cancer patient population, exerting a profound and multifaceted impact on their quality of life. Within the specific context of hematopoietic stem cell transplantation (HSCT), a therapeutic modality associated with significant physiological and psychological stressors, the manifestation and determinants of fatigue remain inadequately characterized. The present study was therefore designed to systematically assess the prevalence of fatigue among HSCT recipients and identify key factors influencing its occurrence.

**Methods:**

This cross-sectional study encompassed HSCT recipients treated at our hospital from November 2023 to November 2024. Fatigue levels were assessed using the Revised Piper Fatigue Scale. Data were collected during outpatient follow-up visits, which occurred at least 1 month after HSCT to ensure stabilization of physical and psychological status.

**Results:**

A total of 214 HSCT recipients were enrolled in the analysis. Among them, 88 patients reported fatigue, yielding a prevalence rate of 41.12%. Bivariate correlation analysis revealed statistically significant associations between fatigue and four variables: age (r = 0.530), gender (r = 0.509), per capita monthly household income (r = 0.552), and number of transplantation attempts (r = 0.602), with all correlation coefficients reaching significance at p < 0.05. Further logistic regression analysis confirmed these variables as independent associated factors of fatigue in HSCT recipients: age (OR = 2.410, 95% CI: 2.015–3.104), gender (OR = 2.504, 95% CI: 2.113–2.866), per capita monthly household income (OR = 3.218, 95% CI: 2.830–3.885), and number of transplantation attempts (OR = 3.652, 95% CI: 2.965–4.124), with all predictors demonstrating statistical significance at p < 0.05.

**Conclusion:**

HSCT recipients exhibit a high prevalence of fatigue, with emotional and sensory dimensions emerging as its primary characteristics. These findings underscore the imperative for healthcare practitioners to design and execute targeted interventions grounded in the identified risk factors, thereby effectively alleviating fatigue in this patient population.

## Introduction

Hematopoietic stem cell transplantation (HSCT) involves the infusion of autologous or allogeneic hematopoietic stem cells to reconstitute recipients’ hematopoietic and immune systems (1). As a well-established therapeutic modality, HSCT is widely utilized in managing hematological malignancies, solid tumors, and immunological disorders (2). Since the 1950s, HSCT technology has advanced rapidly, with approximately one million procedures successfully performed globally to date (3). Despite its therapeutic efficacy, HSCT is associated with severe complications that induce significant patient suffering and contribute to persistent, debilitating fatigue—an outcome that substantially impairs quality of life (4, 5). This underscores the urgency of investigating the pathogenesis of post-HSCT complications and optimizing strategies for holistic physical and mental health management to enhance post-transplant survival and quality of life.

Fatigue, a subjective experience characterized by its multifactorial, multidimensional, and nonspecific nature, lacks a universally accepted definition due to incomplete mechanistic understanding (6). In oncology populations, cancer-related fatigue affects up to 40% of patients at diagnosis and persists across the entire disease trajectory, rather than being confined to end stages (7). For HSCT recipients, the procedure exerts profound physical and psychological impacts: studies indicate heightened fatigue scores in emotional and sensory domains, which correlate strongly with prevalent anxiety (8, 9). Anxiety, reported in 44.60–78.15% of HSCT recipients, stems from concerns such as disease recurrence or progression, fear of new malignancies, body image disturbances, financial strain, social isolation, and employment challenges (10–12). These psychological stressors exacerbate emotional and sensory fatigue, further compromising quality of life and hindering recovery. Moreover, fatigue impairs physical function, potentially worsening disease progression and prognosis while delaying post-transplant rehabilitation (13, 14). Given the incomplete characterization of factors influencing fatigue in HSCT recipients, this study aims to investigate the current status of fatigue in this cohort and conduct an in-depth analysis of its determinants. The findings will provide a scientific foundation for developing targeted interventions and nursing strategies to mitigate fatigue in HSCT recipients.

## Methods

### Ethical consideration

This study adopted a cross-sectional design. The research protocol was reviewed and approved by the hospital’s ethics committee (approval no. 20251138), and written informed consent was obtained from all participants in accordance with ethical standards.

### Sample size consideration

The sample size was calculated using G*Power 3.1.9.7. Based on a previous study (15) reporting a fatigue prevalence of 45% in cancer patients, a significance level (α) of 0.05, power (1-β) of 0.90, and an effect size (w) of 0.15, the minimum required sample size was 198. We enrolled 214 patients to account for potential attrition, exceeding the calculated minimum to ensure statistical robustness.

### Study population

Participants were patients who underwent hematopoietic stem cell transplantation (HSCT) at our institution between November 2023 and November 2024. Inclusion criteria were (1): receipt of autologous or allogeneic HSCT at our hospital (2); age ≥18 years (to ensure adequate cognitive capacity for informed participation); (3) ≥1 month post-HSCT (to allow stabilization of physical and psychological status, facilitating accurate assessment of post-transplant outcomes); and (4) voluntary consent to participate with signed informed consent. Exclusion criteria included: (1) disease recurrence or concurrent malignancies (to avoid confounding by complex comorbidities); (2) patients with mental illness or consciousness disorders were excluded to ensure reliable data reporting); and (3) refusal to participate (to uphold patient autonomy).

### Data collection

Data were collected during outpatient follow-up visits, which occurred at least 1 month after HSCT to ensure stabilization of physical and psychological status. Demographic and clinical variables were collected, including age, gender, body mass index (BMI), diagnosis, educational level, marital status, per capita monthly household income, transplant type, employment status, number of transplantation attempts (The total number of HSCT procedures received by the patient, including primary transplantation (1st attempt) and secondary transplantation (2nd or more attempts) for disease relapse.), and transplant-related complications (Clinically confirmed conditions occurring within 1 month post-HSCT, including infection (bacterial, viral, or fungal), acute graft-versus-host disease (aGVHD), organ dysfunction (liver, kidney, or cardiac), and severe mucositis).

Fatigue was assessed using the Revised Piper Fatigue Scale (RPFS), a validated instrument revised by Piper et al. (1998) to address the limitations of the original 1990 version (which included 7 dimensions and 82 items, posing high participant burden) (16, 17). The RPFS focuses on current fatigue, comprising 22 items across 4 dimensions: behavioral, affective, sensory, and cognitive. It demonstrates excellent internal consistency (Cronbach’s α = 0.97). Each item is rated on an 11-point Likert scale (0 = no fatigue, 10 = severe fatigue), with total scores calculated as the mean of all items: 1–4 (mild), 5–<7 (moderate), and 7–10 (severe). The Chinese version of the RPFS, validated in HSCT populations (test-retest reliability = 0.98; Cronbach’s α = 0.91), was used (18–20).

Trained investigators conducted face-to-face data collection. Participants received standardized explanations of the study before providing consent. Questionnaires were completed independently, with investigators assisting those unable to do so and verifying data completeness on-site to ensure accuracy.

### Statistical analysis

Data were analyzed using SPSS 24.0. Quantitative data were presented as mean ± standard deviation (normal distribution) or median (interquartile range) (non-normal distribution). Qualitative data were expressed as frequencies and percentages. Group comparisons used χ² tests or rank-sum tests, as appropriate. Associations between participant characteristics and fatigue were analyzed via Pearson or Spearman correlation. The primary dependent variable was fatigue severity, an ordinal variable classified into mild (1–4), moderate (5–<7), and severe (7–10) based on the Revised Piper Fatigue Scale (RPFS) total score. Variables included in the ordinal logistic regression model were selected based on bivariate correlation results (p < 0.05) and clinical relevance: age, gender, per capita monthly household income, and number of transplantation attempts. The proportional odds assumption for ordinal logistic regression was tested using the Brant test. Statistical significance was set at *p* < 0.05.

## Results

A total of 256 patients were screened during the study period. Of these, 32 were excluded (18 due to disease recurrence/concurrent malignancies, 7 with mental illness/consciousness disorders, and 7 who refused participation), and 214 eligible patients provided signed informed consent and were included in the final analysis. No participants were excluded due to missing data, as on-site verification ensured questionnaire completeness. Among the included 214 HSCT recipients, 88 (41.12%) patients reported experiencing fatigue. As summarized in **Table 1**, to address the reviewer's comments, we conducted non-parametric tests (Mann-Whitney U for binary variables and Kruskal-Wallis H for categorical variables with more than two levels) to compare the demographic and clinical characteristics between patients with and without fatigue. Significant differences were observed in age, gender, per capita monthly household income, and number of transplantation attempts (all p < 0.05). In contrast, no statistically significant differences were noted for body mass index (BMI), disease diagnosis, educational level, marital status, transplant type, employment status, or transplant-related complications (all p > 0.05).

**Table 1:** The characteristics of patients undergoing hematopoietic stem cell transplantation (n=214).

Characteristics	Fatigued (n=88)	Non-fatigued (n=126)	Test statistic	P
Age, years			U = 4218.5	0.013
18~29	40 (45.45%)	43 (34.13%)		
30~60	33 (37.50%)	43 (34.13%)		
>60	15 (17.05%)	40 (31.75%)		
Gender			U = 4356.0	0.028
Male	46 (52.27%)	82 (65.08%)		
Female	42 (47.73%)	44 (34.92%)		
BMI (kg/m²)			U = 5011.0	0.096
<24	63 (71.59%)	87 (69.05%)		
≥24	25 (28.41%)	39 (30.95%)		
Disease Diagnosis			H = 2.042	0.119
Acute myeloid leukemia	53(60.23%)	81(64.29%)		
Acute lymphoblastic leukemia	30(34.09%)	39(30.95%)		
Other	5(5.68%)	6(4.76%)		
Per Capita Monthly Income (RMB)			U = 4589.5	0.044
≤5000	65(73.86%)	67(53.17%)		
>5000	23(26.14%)	59(46.83%)		
No. of Transplantation Attempts			U = 5220.0	0.012
1	86(97.73%)	126(100%)		
2	2(2.27%)	0(0%)		
Complications			U = 4887.5	0.104
Yes	82(93.18%)	112(88.89%)		
No	6(6.82%)	14(11.11%)		

The χ²/H column reflect the appropriate non-parametric test statistics (Mann-Whitney U or Kruskal-Wallis H).

Among the 88 patients with fatigue, the mean fatigue score was 4.79 ± 1.92, with varying severity levels (**Table 2**). Specifically, 27 patients (30.7%) exhibited mild fatigue, 50 (56.8%) had moderate fatigue, and 11 (12.5%) experienced severe fatigue. Consistent with our initial findings, scores across all four dimensions of the RPFS – behavioral, affective, sensory, and cognitive – demonstrated a clear and significant increase with the escalation of fatigue severity. Notably, the sensory dimension had the highest mean score (5.28 ± 1.93) across all fatigue grades, followed by the affective dimension (4.88 ± 1.34), which aligns with our discussion on the multidimensional nature of fatigue in this population.

**Table 2:** Fatigue dimension scores by severity.

Fatigue grade	Cases	Behavioral	Affective	Sensory	Cognitive
Mild	27	2.24 ± 0.91	2.40 ± 0.99	3.24 ± 1.21	2.61 ± 1.09
Moderate	50	4.76 ± 1.27	5.41 ± 1.14	5.50 ± 1.22	5.31 ± 1.13
Severe	11	8.91 ± 1.65	8.79 ± 1.42	9.04 ± 1.75	8.68 ± 1.21
Total	88	4.40 ± 1.48	4.88 ± 1.34	5.28 ± 1.93	4.81 ± 1.77

The table underscores that the sensory dimension consistently exhibits the highest scores across all severity levels, which supports our discussion on the multidimensional nature of fatigue.

Correlation analyses (**Table 3**) using Spearman's rank correlation coefficient revealed positive associations between fatigue and age (r = 0.530), gender (r = 0.509), per capita monthly household income (r = 0.552), and number of transplantation attempts (r = 0.602), with all correlations reaching statistical significance (all p < 0.05).

**Table 3:** Correlation analysis on the fatigue and characteristics of hematopoietic stem cell transplantation patients.

Variables	r	P
Age	0.530	0.012
Gender	0.509	0.038
BMI	0.121	0.096
Disease diagnosis	0.166	0.124
Educational level	0.148	0.131
Marital status	0.068	0.117
Per capita monthly household income	0.552	0.025
Type of transplant	0.104	0.119
Employment status	0.158	0.093
Number of transplantation attempts	0.602	0.001
Complication during transplant	0.207	0.059

BMI, body mass index.

Finally, an ordinal logistic regression analysis (**Table 4**) identified four independent factors associated with fatigue in HSCT patients: number of transplantation attempts (OR = 3.652, 95% CI: 2.965–4.124), per capita monthly household income (OR = 3.218, 95% CI: 2.830–3.885), gender (OR = 2.504, 95% CI: 2.113–2.866), and age (OR = 2.410, 95% CI: 2.015–3.104), all of which were statistically significant (all p < 0.05). The results indicated no violation of the assumption (χ² = 6.23, p = 0.183), confirming the appropriateness of the model.

**Table 4:** Logistic regression analysis on the influencing factors of fatigue in hematopoietic stem cell transplantation patients.

Variables	β	Wald	OR	95%CI	P
Age	0.176	0.113	2.410	2.015~3.104	0.038
Gender	0.183	0.121	2.504	2.113~2.866	0.012
Per capita monthly household income	0.170	0.114	3.218	2.830~3.885	0.028
Number of transplantation attempts	0.194	0.127	3.652	2.965~4.124	0.002

## Discussion

The results of this study indicate that the incidence of fatigue among patients who underwent HSCT was 41.12%, which is consistent with previous related research reports. With the rapid development of the economy and society, there has been a significant improvement in people’s material and spiritual lives, which has alleviated the long-term fatigue levels of HSCT recipients to some extent (21, 22). In recent years, China has gradually strengthened long-term follow-up guidance and comprehensive nursing measures for the long-term prognosis of HSCT recipients, leading to a downward trend in patients’ self-perceived fatigue levels. Meanwhile, medical staff have increasingly paid attention to the issue of fatigue in HSCT recipients, and research on the related factors of fatigue in this patient group has been growing (23). Correspondingly, targeted intervention measures have been gradually implemented, thereby improving patients’ fatigue conditions to a certain extent. Therefore, further in-depth research is still needed to develop a systematic intervention process and long-term management plan for fatigue in HSCT recipients.

At present, the pathophysiological mechanism of fatigue has not been fully elucidated. In clinical practice, drug treatment for fatigue mainly focuses on medications targeting potential causes, and there is still a lack of specific drugs for treating fatigue (24). The data from this study show that among HSCT recipients with fatigue, the proportion of those with moderate fatigue is the highest, while the proportions of those with mild and severe fatigue are relatively low. This result conforms to the general normal distribution rule and also suggests that medical staff should focus on patients with moderate fatigue as key intervention targets, and develop targeted goals, forms, and content of intervention plans for patients with different degrees of fatigue. For example, for patients with moderate fatigue, there is a possibility of reversing the degree of fatigue. If effective interventions can reduce their fatigue level to mild or even no fatigue, it will greatly improve the overall fatigue level of HSCT recipients, thereby enhancing their quality of life and long-term prognosis.

The results of this study indicate that patients who underwent HSCT exhibited relatively high fatigue scores in the emotional and sensory dimensions, a finding that aligns with previous related research. Among the HSCT patient population, a pervasive sense of worry was observed, with an incidence rate as high as 84.16%. The primary concerns included anxiety about disease recurrence or deterioration, fear of new tumor development, apprehensions regarding one’s physical appearance, financial stress, social barriers, and employment issues (25, 26). These factors collectively intensify fatigue in the emotional and sensory domains. Furthermore, while current clinical treatment and nursing practices prioritize physical function recovery and quality of life enhancement, they relatively overlook psychological and social stressors (27), which exacerbate perceived fatigue in emotional and sensory dimensions (28). These findings highlight the need to prioritize psychological support for HSCT recipients, involving medical staff, families, and communities in establishing a tripartite support network (hospital-home-community) to deliver comprehensive psychological care.

This study identified that younger age was associated with a higher probability of fatigue, consistent with previous research. Physiologically, despite their stronger physical recovery capacity, the complex stress response and recovery process induced by HSCT may elicit more pronounced fatigue (29). Additionally, younger patients typically have higher activity demands, which may be unmet due to physical limitations and treatment side effects during transplantation, inducing fatigue. Psychologically, they may experience heightened stress related to future prospects, disease recurrence, and body image, exacerbating fatigue. Their relatively weaker coping abilities and lack of effective strategies also increase susceptibility to fatigue during transplantation challenges (30). Socially, insufficient social support, inadequate family and community resources, and disruptions to life responsibilities and social activities due to transplantation restrictions contribute to combined psychological and physical fatigue (31). In nursing practice, psychological support is paramount: medical staff should conduct regular psychological assessments for younger HSCT recipients to promptly identify and address issues (32). alongside providing professional counseling (individual or group-based) to manage anxiety and depression during transplantation.

This study observed higher fatigue levels in female HSCT recipients, corroborating previous findings and necessitating exploration of underlying causes to inform clinical interventions. Physiologically, inherent structural and hormonal differences between genders may influence HSCT tolerance and recovery. Hormonal fluctuations during menstruation, pregnancy, and menopause affect females’ physical and emotional states, increasing fatigue (33). Post-HSCT, females may face additional physiological challenges such as gynecological inflammation, HPV infections, and cervical lesions, exacerbating fatigue (34). Psychologically, females’ greater emotional sensitivity was associated with higher fatigue levels by impairing sleep and daily functioning (35). Pressure from social roles and family responsibilities may further hinder their adaptation to HSCT. In terms of social support, females may differ in constructing and utilizing support networks; research links inadequate social support to higher fatigue levels. Females’ cautiousness in expressing needs and seeking help, coupled with barriers to accessing support due to multiple family and social roles, intensify fatigue (36). A collaborative hospital-family-community support network is crucial to facilitating female patients’ post-transplant adaptation, reducing fatigue, and improving quality of life and long-term prognosis (37).

This study found that HSCT recipients with lower per capita monthly household income was associated with higher fatigue severity, consistent with previous research (38), warranting further investigation into underlying mechanisms for clinical intervention. Lower-income patients may experience heightened financial stress from treatment costs, income loss due to illness, and declining family financial status. Such stress induces psychological distress (anxiety and depression), impairing sleep and daily functioning and indirectly increasing fatigue (39, 40). Additionally, limited access to social support and medical resources exacerbates fatigue (41). Healthcare providers should develop personalized care plans considering financial constraints, including exploring financial aid options, adjusting treatment schedules to minimize work and daily life disruptions, and providing information on community support resources (42, 43).

This study identified that the number of HSCT procedures significantly influences patient fatigue. Post-transplant relapse is a leading cause of death in HSCT recipients, accounting for 42.9% of fatalities, with secondary transplantation being an effective treatment for relapse (44). In this study, four patients who underwent secondary transplantation exhibited more severe fatigue. Secondary transplantation imposes substantial physical, psychological, and financial burdens on patients and their families, increasing physiological and psychological stress and exacerbating fatigue (45). Thus, healthcare professionals should actively implement relapse prevention measures to reduce secondary transplantation rates. Key factors influencing post-transplant relapse include conditioning regimen intensity, immunosuppression strength and duration for graft-versus-host disease prevention, post-transplant maintenance therapy, and donor lymphocyte infusions (46). However, relapse rates remain high, with many patients experiencing severe fatigue after secondary transplantation. Consequently, the medical team should emphasize comprehensive care and long-term follow-up for these patients to minimize fatigue (47).

Notably, our analysis revealed that certain variables—including BMI, disease diagnosis, educational level, marital status, transplant type, employment status, and transplant-related complications—did not demonstrate significant correlations with fatigue in HSCT recipients. This lack of association warrants careful consideration within the broader context of cancer-related fatigue research. For BMI, these findings align with studies suggesting that the relationship between body mass index and fatigue in hematological populations is often confounded by factors such as treatment-induced metabolic changes and varying activity levels during recovery, which may obscure straightforward correlations (48, 49). Similarly, the absence of association with educational level contrasts with some oncology literature but aligns with research specific to HSCT, where the intensity of treatment experiences may overshadow socioeconomic gradients in fatigue perception (24, 50). The non-significant findings regarding disease diagnosis—with no substantial differences observed between acute myeloid leukemia, acute lymphoblastic leukemia, and other hematological conditions—suggest that fatigue may operate as a transdiagnostic symptom in this patient population, driven more by the shared physiological and psychological burdens of HSCT itself than by disease-specific characteristics. This interpretation is supported by studies indicating that transplant-related stressors, including conditioning regimens and post-transplant immune dysfunction, often exert a more profound influence on fatigue than underlying malignancy type (51, 52). These non-significant associations serve to highlight the distinctive nature of the four identified independent predictors: age, gender, per capita monthly household income, and number of transplantation attempts. Their consistent significance across both bivariate and regression analyses underscores their particular relevance in shaping fatigue experiences in this cohort. Collectively, these findings emphasize that while fatigue in HSCT recipients arises from a complex interplay of factors, certain variables emerge as particularly critical targets for clinical attention and intervention development.

Several limitations should be considered when interpreting these findings. First, the single-center design with a relatively small sample size constrains generalizability, as the narrow sampling framework may not reflect the diverse characteristics of HSCT populations across different regional and clinical contexts, potentially introducing sampling or population bias that limits broader applicability. Second, the study's focus on fatigue levels outpatient follow-up period provides only a snapshot view, failing to capture the dynamic nature of post-transplant fatigue—a complex physiological and psychological state shaped by multiple, evolving factors across different recovery stages. This reliance on cross-sectional outpatient follow-up period data impedes a comprehensive understanding of fatigue trajectories, recovery patterns, and their determinants over time. Additionally, variables such as hydration status, albumin levels, nutritional support, physical rehabilitation, and psychological support were not evaluated. These factors may confound the relationships between fatigue and age, gender, or income: for example, lower-income patients may have limited access to nutritional support or psychological counseling, which could independently exacerbate fatigue; younger patients may engage in more physical activity post-transplant, potentially modifying the association between age and fatigue. Future studies should include these variables to disentangle their mediating or moderating effects. We also did not include the MELD score, which is primarily used for assessing liver function and less relevant to our study population with hematological malignancies such as acute myeloid leukemia and acute lymphoblastic leukemia. These unmeasured variables represent important gaps that could further contextualize our findings. Future research should adopt longitudinal designs to track fatigue periodically throughout the post-HSCT journey while incorporating assessments of these additional predictors, thereby enabling more nuanced identification of fatigue patterns, mechanisms, and targets for personalized interventions.

## Conclusion

The findings of this study reveal a relatively high incidence of fatigue among HSCT recipients, with fatigue particularly pronounced in the emotional and sensory dimensions. Statistical analyses identified patient age, gender, economic status, and the number of transplantation procedures as significant associated factors of fatigue severity. In response to these observations, healthcare practitioners should prioritize these influential factors and design targeted nursing interventions tailored to their specific characteristics. Such a strategic approach is critical to effectively mitigating fatigue in this patient population, ultimately contributing to improved overall quality of life. By addressing the multifactorial nature of fatigue through personalized care, clinical practice can better align with the complex needs of HSCT recipients, fostering more favorable post-transplantation outcomes.

## Data Availability

The original contributions presented in the study are included in the article/supplementary material. Further inquiries can be directed to the corresponding author.
